# Perceptions of health and illness among the Konso people of southwestern Ethiopia: persistence and change

**DOI:** 10.1186/s13002-018-0214-y

**Published:** 2018-02-26

**Authors:** Tebaber Workneh, Guday Emirie, Mirgissa Kaba, Yalemtsehay Mekonnen, Helmut Kloos

**Affiliations:** 1 Department of Social Anthropology, University of Gondar, PO BOX 196, Gondar, Ethiopia; 2 Department of Social Anthropology, AAU, PO BOX 1196, Addis Ababa, Ethiopia; 3 Department of Public Health, AAU, PO BOX 1196, Addis Ababa, Ethiopia; 4 Department of Biology, AAU, PO BOX 1196, Addis Ababa, Ethiopia; 5 Department of Epidemiology and Biostatistics, University of California, San Francisco, CA USA

**Keywords:** Indigenous medicine, Illness perceptions, Health-seeking behavior, Konso ethnic group, Ethiopia

## Abstract

**Background:**

Cross-cultural studies indicate that every culture has its own particular explanations for health and illness and its own healing strategies. The Konso people have always practiced indigenous medicine and have multifaceted accounts or multiple dimensions of illness perceptions and health-care beliefs and practices. This paper describes how perceptions of health and illness are instrumental in health and treatment outcomes among the Konso people in southwestern Ethiopia. Results may provide an understanding of the perceptions of health and illness in relation to the local cosmology, religion, and environment.

**Methods:**

The ethnographic method was employed to generate evidence, complemented by focus group discussions, in-depth interviews, and direct observation. Thematic analysis was employed to categorize and interpret the data.

**Results:**

Findings indicate that the Konso people’s worldview, particularly as it relates to health, illness, and healing systems, is closely linked to their day-to-day lives. Older people believe illnesses are caused by a range of supernatural forces, including the wrath of God or local gods, *oritta* (spirit possession), and *karayitta* (ancestral spirits), and they use culturally prescribed treatment. Young and formally educated members of the community attribute causes of diseases to *germitta* (germs) and *factorta* (bacteria) and tend to seek treatment mostly in modern health facilities.

**Conclusion:**

Perceptions of health and illness as well as of healing are part of Konso people’s worldview. Local communities comprehend health problems and solutions within their cultural frame of reference, which has changed over the years. The Konso people associate their health situations with socio-cultural and religious factors. The individual’s behavior and interactions with the social, natural, and supernatural powers affect the well-being of the whole group. The individual, the family, the clan leaders, and the deceased are intimately linked to one’s culturally based health beliefs and are associated by the Konso with health problems and illnesses.

## Background

The Konso worldview regarding health, illness, and health-care systems is closely linked to the culture of the people and their knowledge of the natural environment, plants, and animals. This relationship is corroborated by Hallpike [1], who noted that the Konso’s worldview symbolizes the belief system that is embedded in both the physical and spiritual worlds and in the metaphysical forces of the universe. This study presents the Konso people’s understanding of health in relation to local customs, religion, and environment. Various studies indicate that health and illness perceptions can elucidate people’s health-care options and health-seeking behaviors [1–6]. Ethno-medical beliefs and practices continue to be widely evident in Ethiopia [7–14]. Their persistence indicates considerable cultural continuity and persistent under-utilization of most biomedical health services [15]. Healers frequently combine magico-religious rituals and empirical techniques, usually including medicinal plants and holy water (*tsebel*), in their treatment strategies [16–18].

The Konso cosmology is closely associated with their worldview and belief system [19]. According to their worldview, the universe is divided into three worlds: *waaqa* (the sky/heaven), *pita* (the earth), and *xatikela* or *pitakela* (the underworld) [20]. Apparently, the Konso have a myth according to which there was in the beginning a gourd planted by *waaqa* that grew until it burst, forming various people: *ettanta* (farmers), *xawda* (craftsmen), and *poqqallada* [21]. This cosmology reveals that the Konso beliefs about their origin are associated with natural resources and the environment. During our fieldwork, we observed and perceived the implications of the cultural cosmology in different dimensions. The cosmology is closely linked to perceptions of the Konso regarding the cause of illness and strongly influences their health-care beliefs and practices.

In the absence of medico-anthropological studies among the Konso, little is known about their perceptions of health and illness and their treatment of health-related conditions. In this paper, we describe and analyze the cultural perceptions and practices surrounding health and illness in the Konso community.

The Konso people maintain various socio-cultural values that bear on illness and health-seeking behavior. According to Hallpike [21, 22], the Konso consider their cultural values to be very important for their communal life. In this study, we found that trustworthiness, self-respect, hospitality, hardwork, pride in their identity, and confidence are prominent cultural values of the Konso.

Both indigenous and biomedical health-care services are available to the Konso. The indigenous health-care services include spiritual/religious healers (*ahaburandininno*), herbalists (*harimmitta* or *appaqoracha uppo*), bone setters (*shemabiyta*), and traditional birth attendants (*shorrambyta*). People in the study area reported that most health posts and private clinics lack medical equipment, drugs, and trained and skilled manpower. As a result, for several diseases, the people use indigenous healers or treat their health problems themselves. The deficiencies in health-care services contribute to the use of indigenous healers and home treatments.

## Methods

The study was carried out in Konso *Woreda* (District) from February 2012 to March 2013. Konso *Woreda* is located in southwestern Ethiopia, about 600 km southwest of Addis Ababa and 360 km from the regional capital, Hawassa [11, 23]. According to the Statistical Report of the Population and Housing Census [12], the Konso population in 2007 was 234,987, of which 113,353 (48.2%) were males and 121,634 (51.8%) females.

### Study design

The study employed a qualitative explorative design. The various types of information related to perceptions and personal meanings of indigenous medicine were collected using in-depth interviews, focus group discussions (FGDs), and direct observations in the field.

### Data collection

Throughout our fieldwork, the principal investigator (TC) played the role of systematic observer of occasions, practices, and activities. We observed the physical and biotic environment, medicinal plants, herbal markets, *mora* (public centers), farmlands, sacred forests, biomedical health centers, and village compounds to understand the social relationships among the people and enablers in the medical system. Observations were recorded as mental and written notes and photographs for later recall. Observations of day-to-day activities of the people helped to contextualize how they perceived and conceptualized illness, the traditional treatment system, and health-seeking behaviors.

In qualitative interviews, knowledge is produced through conversations between the interviewer and the interviewee on certain themes [24]. We carried out in-depth interviews with 44 informants of both genders from different age, occupational, residential, and educational groups about their health and beliefs and perceptions regarding illness and treatment. We interviewed 12 key informants: 6 herbalists (2 females and 4 males), 3 local elders, 1 religious healer, and 2 indigenous medical beneficiaries who had direct and indirect exposure to the use of indigenous medicine. We interviewed additional 20 informants from other occupational groups, mostly farmers. We initiated the discussions by asking questions from different interview checklists specific to the groups. The interviews were voice-recorded with the approval of the interviewees.

The use of key informant interviews is desirable because “key informants have been thoughtfully and purposefully chosen for the knowledge they have about the culture under study, while any [other] informants may not be fully knowledgeable” [25]. In-depth interviews provided informants with the opportunity to explain their experiences and behaviors related to indigenous medicine. They encouraged people to talk freely about their personal feelings, opinions, and perceptions and facilitated our understanding of the meanings of symbols, ritual interactions, and personal meanings of indigenous medicine. This method enabled us to understand knowledge-related issues in illness causation, indigenous beliefs surrounding health and ill-health, and personal explanations of individual episodes of illness.

Focus group discussion (FGD) is another qualitative method used in this study. According to Casley and Krueger [26, 27], FGDs involve a carefully selected group of people with certain common interests and similar levels of experiences in group discussions on broad public issues. FGDs were held to help us grasp the different and shared attitudes and perceptions of the participants with respect to indigenous medicine. FGDs were instrumental in the triangulation [26] of the findings to provide a broader understanding of Konso health and illness perceptions. By and large, this method helped us elaborate on and exemplify issues explained by the other data gathering methods used.

With the assistance of the research interpreters, we purposefully selected FGD participants with similar demographic and socio-cultural backgrounds. Our prior exposure to people during our informal interviews and discussions made the FGD recruitment easier. A total of 18 FGD sessions were arranged for groups selected on the basis of common interests and experiences. At the 18th FGD session, the data reached saturation level as the information obtained in previous sessions recurred repeatedly. Participants in the 18 FGD sessions were elderly men, elderly women, and younger men and younger women representing indigenous healers, local communities, agricultural development agents, and female health extension workers. Three FGDs were held with indigenous healer groups (1 mixed-gender, 1 male, and 1 female group). Another male FGD group included agricultural development agents. We also conducted 3 FGD sessions with health extension workers in Karat town. In each of the 5 *kebeles*, we held at least 2 FGDs with mixed or separated male and female participants from the local community. The number of people participating in each FGD session ranged from 6 to 12. For each FGD session, it took on average 2 ½ h. The lead author and his assistant organized and managed the FGD sessions and served as both moderators and note takers. A total of 108 men and women living in the study villages participated in FGDs.

### Data analysis

The information obtained from the FGDs and intensive interviews was recorded with the approval of the study participants and transcribed from *Konsoigna* to English and analyzed thematically. Various themes were formed based on issues such as perceptions about health and illness and perceived causes of illness. Differences in the initial analysis were resolved and different categories agreed upon. The various categories were then used to code the transcription for each participant’s responses. Subsequently, transcriptions and coding were translated into English by a coder who was familiar with the research objectives to ensure the reliability of the codes. Likewise, we developed codes to categorize the information into important themes based on the specific objectives. We used “theme analysis techniques” such as Coding (i.e., topic and analytical coding). Topic coding here helped us to gather materials, and analytical coding was used to generate new ideas. For example, it helped to check ideological assumptions, contradictions, omissions, turning points, ambivalence, etc. Coding was done manually. It was done by using color pencils, folders, and cards. Due to the diversity of the qualitative responses, it was necessary to use theme analysis techniques to interpret the data and identify common themes. Thematic analysis was then employed to analyze, and at times synthesize, the main topics of categorized data gathered through systematic observation, in-depth interviews, and FGDs. This method served to identify systematic patterns or relationships among categories. It also helped us to better comprehend the symbolic meanings of the local beliefs and health practices.

### Data quality assurance

The different types of information gathered help to explain human worldviews and elucidate the emic, or insiders’, perspective rather than focus merely on the etic, or outsiders’, perspective. We used various criteria to assure quality of the qualitative data. Some of the criteria were trustworthiness, credibility, and conformity [24]. We tried to ensure trustworthiness and credibility by triangulation with multiple sources of data collection methods, thereby enhancing the validity of the study. We also tried to ensure the credibility and representativeness of the research findings through confirmation by knowledgeable research participants and language interpreters. We evaluated the information by examining whether the study results were representative of Konso culture and lifestyle. Therefore, it was possible, in the end, to ensure that this interpretation would correctly reflect the research participants’ perceptions of health and illness.

## Results

### Understanding health and illness

Beliefs about health and illness were reflected in the day-to-day lives of the Konso people. Data from observations indicate that the concept of health for the Konso people goes beyond the idea of well-being. To be healthy not only is to be well and free from illnesses but also is manifested by proper eating, drinking, and social interaction and performing local ceremonies as part of the community. Informants explained that health for the Konso encompasses receiving enough rain during the rainy season, having good harvests, and living harmoniously with the environment, *waaqa* (*the indigenous god*), and *karayitta* (ancestors). The Konso people usually express the notion of health while greeting each other by saying *fayankeakamii*, which means “how is your health condition?”

A Konso person may become ill or die if he/she gives false witness in front of a *poradukata* (the erected stone or place of truth in the town center). A ritual known as *moora dawra* (place of truth, where dead human beings are believed to be still living) plays a central role in purifying victims of disease after stealing or lying. According to a 23-year-old male informant, if two people are in conflict and go to the clan leader and accuse each other, they are expected to tell the truth in front of the clan leader. If one is lying, he/she will be punished through *xaxa* (oath), which is believed to cause madness, illness, or death of the entire family of the cursed individual via the curse of the *poqqallada*. This is similar to the curse known as *hada*, a spiritual power that can harm culprits. The *poqqallada, who* act as priests, play a powerful role in illness prevention. They frequently perform rituals and pray to their *waaqa* to protect people from illnesses and various catastrophes. Two FGD participants explained that the *poqqallada* play the role of guardians of the local environment and traditions.

Social codes and the power of words such as *jija*/*chicha* (curses) and *fayasifiakenu* (may God give you health, i.e., bless you) are considered important in shaping people’s perceptions of health and causes of ill-health. In Konso culture, some inherited social codes include cultural norms prescribing individual and social behavior. Curses by the *poqqallada* and local elders are examples of the power of words in Konso. For example, if one marries a clan member, clan leaders curse him/her to be unproductive and have a short life. The curses may also cause illness.

The Konso perceive the environment outside the home to be dangerous for vulnerable groups. Data obtained from a 62-year-old male informant and 10 mixed-gender FGD participants indicate that if a newly born child is seen by people other than traditional birth attendants, that child is believed to be affected by the evil eye, or the devil’s spell. Moreover, when a pregnant woman becomes ill, it is forbidden to take the woman to biomedical health-care practitioners. According to a 23-year-old female health extension worker, this perception prevents mothers from bringing their newly born children to clinics for vaccinations and treatment and results in widespread childhood infectious diseases.

The relationship between biomedical and Konso indigenous health practitioners is complicated and often results in negative health outcomes. Although interviews with health-care providers did not reveal negative attitudes towards *shorrambyteta* (traditional birth attendants), some of the health extension workers complained about their practices. The health extension workers believed the *shorrambyteta* negatively affected the health of children and mothers by treating them unsuccessfully and thus delaying trips to the biomedical health services.

The custom of polygamy is a challenge in the prevention of sexually transmitted diseases, including AIDS, and a cause of conflict in gender relationships. Respondents stated that males whose wives do not bear sons to whom they can pass on their wealth upon their death try to impregnate their concubines. This practice is maintained by cultural norms and the patriarchal kinship system, which socially elevates men who are polygamous and have male children.

### Perceived causes of illness

Most illnesses in Konso are believed to be caused by either supernatural forces or the natural environment. Many older, poorer, and less-educated people attributed illness to the wrath of supernatural and malevolent spirits, envious witchcraft, or intrusion of pathological agents as discussed below.

### Supernatural causes of illness

According to key informants, the Konso people conceptualize illness causation as a misfortune attributed to the wrath of God or gods, sorcery, witches, the actions of spirits, and failure to observe taboos. Data obtained from the in-depth interviews revealed that some ailments are believed to be caused by malevolent spirits. People in Konso believe that *saytana*, *shetana*, or *oritta* (devil or evil spirits) are the major causative agents of illnesses such as mental illness and madness. The Konso prefer to treat such health problems locally, by indigenous healers. Informal discussions revealed that people in Konso believe that traveling alone in rural areas at night or at noon can result in afflictions caused by evil spirits because these are times when evil spirits roam around.

Afflictions caused by *karayitta*or *ekera* (spirits of ancestors) are, according to mixed-gender FGD participants, results of the failure to observe taboos—for example, defying clan leaders’ and parents’ orders, neglecting the customs of the community, or not attending community rituals. In one village, participants in a male FGD agreed that spirits of deceased ancestors work with living humans, either for protection or discipline. Most of the seven mixed-gender FGD participants believed ancestors are the major forces of illness causation in their community.

Ancestors were linked to health-related explanations and symbolic interpretations and were believed to cause or prevent illnesses. Most of the interviewed healers believed failure of the living to maintain their traditions may upset the ancestors, who bring misfortune to noncompliant descendants. A 62-year-old male informant from Gamole Village explained that angry ancestors had caused individuals to be born with ahead ancestor inside them because the family had either not held a ritual ceremony or had performed it improperly. Other participants explained that ancestors do not always bring misfortune to the living and that ancestral spirits can be instrumental in leading those suffering from illness to indigenous healers to be healed.

According to key informants, many people believe there is life after death and an ill person who is about to die communicates or speaks with ancestral spirits (*kaarayya*). Information obtained from an in-depth interview revealed a belief that when the spirits are dissatisfied with sacrifices and ritual ceremonies, or when people take wrong actions such as shedding blood of clan members, *qayya* (a group of spirits) and *awuda* (epidemic diseases) invade communities, causing illness and death. To ease the wrath of these spirits, the Konso people hold a *killana* (a special ritual ceremony). Most participants reported that their ancestors were living in harmony with nature, and therefore, they had no health problems. However, they said, because of today’s distortion of culture through modernization, several unknown illnesses were prevalent in the community.

Data obtained from key informant interviews also indicated that *ella* (spirits dwelling in water, springs, or sacred forests) are believed to cause illness among the people of Konso. The perception about the major reason *ella* cause sickness is that people break rules and regulations pertaining to water and sacred forests. Such rules include keeping streams and other waters clean, not entering springs with shoes, preventing cattle from entering water, and not cutting wood from forests without the permission of the *poqqallada*. Breaking these rules was said to cause *tarota* (madness) in wrongdoers or drought. Transgressors are believed to become mad or seriously sick. Local proverbs reinforce these beliefs. One proverb warns, *bekkeessaa qorootte isheetaa taarota keennaado*; literarily, collecting firewood from sacred forests brings madness.

Information obtained from male FGD participants in Jarso Village indicates that ailments attributed to magical or supernatural causes are widespread in the lowlands and around ponds and sacred forests. Therefore, resorting to exorcism and sacrifice with different ritualistic ceremonies as means of curing and protecting from an illness is fairly common in rural areas. Similarly, there is a belief that walking alone in the lowlands or going to fetch water immediately after giving birth can cause madness or death. In an interview, an elder explained that *oritta* (evil spirits) may possess people when they visit lakes and lowland areas at inappropriate times. These spirits attack the family and may possess the children and make them sick. Families can protect themselves and reconcile with the *oritta* by slaughtering a goat or sheep, playing drums, and making offerings to these spirits. A 42-year-old woman in Ollanta Village said the following:

One day, I was going alone to a lowland area for work. Suddenly someone met me and at once I was so sick with severe headache and fell down. When people found me, they brought me back home and on the same day they took me to the diviner’s home. At that time, I was unconscious and did not know what was going on. At another time, after a year, my elder brother was cutting trees in the field and after he came back home, he became very ill and blood flowed from his mouth and nose. We called a diviner from Dokatto and ordered him to perform different rituals by my family to the spirit, including roasting maize and putting it outside the home, making coffee and spike it before it had been tasted. Also different prayers were performed. Unfortunately, my brother could not be cured from his illness and died after three days. So, I believe that the spirits attack not only during night time but also at midday.

Sometimes illness was believed to have been caused by being unclean; this idea was especially common among women. One female informant recounted one experience as follows:

Once I went alone to our lowland farm to collect fire wood. At that time, I was on my menstrual cycle. Two days after I came back from the field, I became ill for about three months. I suffered from severe headache, diarrhea, and vomiting. I was not able to eat well. I only drank coffee and water. Sometimes, I ate roasted grains. After the first two months, I dreamed that a red snake with long hair came to my bed and I was crying as the snake threatened to kill me. I said the snake is coming to poison me and I was afraid. My parents woke up and asked me what had happened. They suspected that the snake is a symbol of ella and that it was the cause of my severe illness. Then, my family made a ritual ceremony to offer blessings to the spirit and after a week, I recovered from my illness.

It is also believed in Konso that if there is a bad relationship between *oritta* (evil spirits), *soayetta* (witches or sorcerers), and a community, these *oritta* and *soayetta* will cause illness in the community. A 22-year-old informant explained that the curses of sorceresses and witches are believed to cause drought, illnesses or diseases, and misfortune. Sometimes people consult the *soayetta* to find out what kind of social problems they will encounter. The predictions of the *soayetta* are communicated through the elected elders, who meet together and discuss the matters at the house of *apa timpa* (the elected community leader) in the village. Data obtained from farmer FGD participants revealed that health-related cultural beliefs and perceptions varied for different illnesses. The issue of supernatural versus natural forces in disease causation is discussed below.

### Naturalistic causes of illness

The educated members of the community associated illnesses and diseases, including headache, tuberculosis, and malaria, with the malfunctioning of organs or the presence of specific disease-causing agents. A few young and educated community members attributed some of the diseases to natural or environmental causes, such as germs, contaminated water, and food shortage. One of the health extension workers and most Konso interviewed in rural areas believed that going to forest and desert areas and eating sorghum cause malaria. In arguing against such perceptions, a 64-year-old man was convinced that when a person goes to the lowlands for farming and remains there for some time, she/he will be exposed to the heat of the sun and sweat a lot, causing dryness of the body and, eventually, malaria. Younger people associated malaria with mosquitoes.

Health and illness perceptions varied along gender lines. Some females maintained that women today are more aware and have more information on causative factors of disease than men because females have greater access to health extension workers and health posts since they are involved with them in family planning education and practices, vaccinations, and birth attending, among other activities. Most men, by contrast, usually spend their time in the fields, although some men were said to have better information and awareness of health-care issues than women because they are in the public arena, where they obtain health information.

Mostly young informants and civil servants thought certain illnesses occur due to specific natural agents such as worms, insects and animals, inherently unhealthy environments, rapid changes in climate, exposure to excessive heat or cold, eating spoiled food or an unbalanced diet, hard work, malfunctioning of specific organs, and heredity. Similarly, civil servant FGD participants believed most diseases were caused by natural agents. The majority of the participants perceived illnesses as being caused naturally. They believed disease can be caused by infection from bacteria (*factorta*) and germs (*germitta*) acquired as a result of lack of proper sanitation. However, they pointed out that they ate their food without washing their hands and did not contract illness because of that practice. Such perceptions *hada* stronghold in rural areas probably because people in towns are more exposed to health education messages than rural residents. Hence, the study indicates that conceptualization of illness causation and symptom presentation in Konso is influenced by changing socio-cultural and demographic circumstances.

## Discussion

This study revealed different conceptions towards health problems and illnesses among the Konso. According to Marshall [28], conception is the process by which information is gathered and interpreted, and it is central to the analysis of health and illness as social phenomena. The role of social prohibitions and taboos and the consequences of their violation with reference to restrictions on eating some taboo foods and disobeying the clan leader is featured strongly in Konso health beliefs. Prohibitions protecting the ecological values of the Konso and the health and environmental consequences of transgression are particularly relevant in Konso *Woreda*, which has experienced environmental degradation and severe drought in recent years.

The findings of this study are consistent with reports of similar beliefs in other African societies. For example, the Akan in Ghana believe illness is a supernatural phenomenon with a magical, superstitious source. They believe violation of cultural norms causes illness [29]. The existence of this belief and its attendant practices in a cultural group can often be best explained by the social fabric of the group’s culture and the environment [30]. The Konso have retained their social and cultural cohesion through local belief systems, ecological values, customs, clanship, and ancestor worship. Their perceptions related to health and illness etiology, like those of the Akan society, are best understood when subsumed under the domains of religion, clanship, and cultural norms [29].

In African societies, many believe illnesses to be caused by psychological or spiritual forces related to the cosmologies of those societies [29, 31]. According to Kleinman [32], every culture has its own particular explanations for health and illness and its own culturally appropriate treatment approaches. In Konso culture, it is believed that if the community neglects to perform the ritual ceremony for a dead *poqqallada*, or if it permits intra-clanship marriage, misfortune including illness and death might come to the community. This belief is also manifested in the curse known as *hada*, by which a spiritual power can harm transgressors. Each individual is thus responsible not only for him/herself, but also for the well-being and equilibrium of the community. These illustrations show the principle of kinship, and its symbolic meaning is believed to directly impact the health of the Konso.

The findings of this study indicate that although local beliefs and cultural traditions continue to dominate the health culture of the Konso, biomedicine is increasingly making inroads. The inroads were revealed in the increasing knowledge of modern medicine among younger people and professionals and changing attitudes towards indigenous healers, including the traditional birth attendants (*shorrambyteta*). The lead author observed that although health extension workers have been trying to change the attitudes of the community towards the use of biomedical health services, the local community still tends to favor indigenous treatment for most health problems. These findings have practical implications for the development of culture-friendly health promotion messages and health services in Konso *Woreda*.

The conceptualizations of health and ill-health by the Konso are in line with Kleinman’s [33] explanation of good health and good fortune as rewards for good behavior, primarily consistent sacrifice to the ancestors, and ill-health and misfortune as punishment for the omission of prescribed sacrifices [32, 33]. Many study participants reported that the major cultural belief system of the Konso is governed by the concept of *ooyritta* (local rules of lifestyle), which is used to predict whether a new year will be happy, healthy, and prosperous or if there will be misfortune, drought, epidemics, and other difficulties. If the coming year is predicted to be full of diseases, sorcerers will advise the elders to perform rituals to forestall the diseases. These conceptualization and practices are consistent with Erinosho’s [34] and Erinosho and Oke’s [35–37] description of illness as a mystical consequence of actions of gods and upset ancestors.

The Konso people’s worldview is central to an understanding of their indigenous healing system, and their perceptions are closely linked to the local cosmology and traditions. According to Kleinman [33], perceptions regarding health and illness strongly influence people’s health practices. Kleinman’s model for explaining behaviors regarding health and ill-health, which is based on patients’ feelings about causes of illness, is reflected in this study. Indigenous Konso healers and their patients have frequently interpreted illness by attributing the origins of disease and/or illness to the cultural, natural, social, and environmental context of the sufferer. A social constructionist paradigm is more appropriate for understanding indigenous healing and medicine. Therefore, when linked to the theoretical framework of this study, the perceptions of causes of illness among the Konso do not substantiate Murdock’s [38] model of personalistic-naturalistic discourse in explaining illness causation.

Relationships between the living and their ancestors similar to those described here as maintained among the Konso have been observed throughout Africa [10, 39]. The perceptions of illness causation summarized above indicate that Konso society can be characterized by a synergy of beliefs and practices regarding the human body and co-existence with other human beings, nature, and spiritual beings. These findings imply that perceptions of health and illness are amenable to increasing health-care utilization. Thus, interventions may influence illness and treatment perceptions to improve associated health outcomes.

## Conclusion

This first study of health and illness within the context of the Konso worldview and beliefs and the ideological level of culture can provide a basis for a broader examination of the link between culture and health behaviors. The Konso health-care system is embedded in local customs and environmental and religious worlds. Health among the Konso is conceptualized holistically, as an integral (mind-body-spirit) phenomenon related to the physiological, psychological, social, and cultural well-being of the individual and the community.

The Konso people associate their health situations with socio-cultural and religious factors. The individual’s behaviors and interactions with the social, natural, and supernatural powers affect the well-being of the whole group. The individual, the family, the clan leaders, and the deceased are intimately linked to one’s culturally based health beliefs and are associated by the Konso with health problems and illnesses. Konso health and illness perceptions are closely linked to symbolic meanings of healing through rituals.

Some apparent inconsistencies were found in the reported perceptions of the Konso. For example, some people viewed particular illnesses, such as malaria, as caused by both naturalistic and supernatural factors. The inconsistencies appear to have resulted from two factors: the retention of traditional values and recent exposure to modern health services and formal education. These factors were reflected in age and social differences among respondents, particularly level of education. When linked to the theoretical framework of this study, the perceptions of causes of illness among the Konso do not substantiate Murdock’s [38, 40, 41] model of personalistic-naturalistic discourse. Kleinman’s [31] explanatory model, however, which is based on patient perceptions of the causes of illness and treatment options, may be useful for systematizing the interpretations of illness of indigenous Konso healers and their patients. Kleinman’s model offers an understanding of Konso health perceptions and behavior that may enable health-care workers to overcome the persistent sharp differences between indigenous and biomedical health services and thereby increase access to modern health services.

If primary health workers are to identify, prevent, and treat illnesses and diseases among the Konso, they must understand the complexities of Konso health knowledge. Understanding the Konso worldview and beliefs and the ideological level of their culture may facilitate the development of a template for addressing cultural impacts on health behavior. This knowledge may allow local health services to incorporate in their operations Konso health and illness perceptions in an integral (mind-body-spirit) manner to promote the physiological, psychological, social, and cultural well-being of the Konso. In addition, the development of culture-specific and culture-friendly health services may result in greater acceptance of biomedicine by the Konso.

## Abbreviations

AAU: Addis Ababa University
AIDS: Acquired immune deficiency syndrome
FGD: Focus group discussion
GE: Guday Emirie
HK: Helmut Kloos
MK: Mirgissa Kaba
TC: Tebaber Chanie Workneh
UOG: University of Gondar
YM: Yalemtsehay Mekonnen
